# Diffuse myocardial fibrosis by post-contrast T1-time predicts outcome in heart failure with preserved ejection fraction

**DOI:** 10.1186/1532-429X-15-S1-M6

**Published:** 2013-01-30

**Authors:** Beatrice A Marzluf, Diana Bonderman, Caroline Tufaro, Stefan Pfaffenberger, Alexandra Graf, Martin Hülsmann, Irene M Lang, Richard Pacher, Gerald Maurer, Julia Mascherbauer

**Affiliations:** 1 Cardiology, Medical University of Vienna, Vienna, Austria

## Background

Diffuse myocardial fibrosis plays a key role in disease progression of heart failure with preserved ejection fraction (HFPEF). Recently it was shown that diffuse myocardial fibrosis is strongly related to post-contrast longitudinal relaxation (T1) time by cardiac magnetic resonance imaging (CMR). The aim of our study was to assess diffuse myocardial fibrosis by CMR T1-mapping in HFPEF patients and test its predictive value.

## Methods

HFPEF was defined as serum NT-proBNP levels > 220 pg/ml, E/e by echocardiography ≥8, signs or symptoms of heart failure and preserved left ventricular ejection fraction (EF≥50%).

63 HFPEF patients and 37 controls were prospectively evaluated. All patients underwent right heart catheterization. CMR studies included the assessment of cardiac function and dimensions by standard cine sequences. Myocardial T1-mapping was performed 15 minutes after a gadolinium bolus using an inversion recovery sequence.

## Results

Post-contrast T1 was significantly correlated with variables reflecting left ventricular filling pressure (E/e p=0.001, R=-0.33; left atrial size, p=0.008, R=-0.27) and pulmonary vascular resistance (p=0.004, R=-0.36).

Patients were followed for a median (range) of 12.9 (0.5-23.1) months. By Kaplan-Meier analysis, event-free survival was significantly worse in patients with T1-times below the median of 388.2ms (log rank p=0.007). By multivariable Cox regression including baseline characteristics, invasive hemodynamics, renal function, and CMR imaging variables, only post-contrast T1 time (p=0.015) and left atrium area (p=0.029) remained independent predictors of event-free survival.

## Conclusions

Our data suggest that post-contrast T1-mapping is a promising tool for the assessment of diffuse myocardial fibrosis. It is closely linked to variables reflecting impaired left ventricular relaxation and hemodynamic variables like pulmonary vascular resistance. Post-contrast T1 time in HFPEF patients outperformed invasive hemodynamics in the multivariable analysis as an independent predictor of event-free survival.

## Funding

none

